# Local Versus Long‐Range Diffusion Effects of Photoexcited States on Radiative Recombination in Organic–Inorganic Lead Halide Perovskites

**DOI:** 10.1002/advs.201500136

**Published:** 2015-07-14

**Authors:** Milan Vrućinić, Clemens Matthiesen, Aditya Sadhanala, Giorgio Divitini, Stefania Cacovich, Sian E. Dutton, Caterina Ducati, Mete Atatüre, Henry Snaith, Richard H. Friend, Henning Sirringhaus, Felix Deschler

**Affiliations:** ^1^ Cavendish LaboratoryUniversity of CambridgeJJ Thomson AvenueCambridgeCB3 0HEUK; ^2^ Department of Materials Science and MetallurgyUniversity of Cambridge27 Charles Babbage RoadCambridgeCB3 0FSUK; ^3^ Department of PhysicsClarendon LaboratoryUniversity of OxfordParks RoadOxfordOX1 3PUUK

**Keywords:** excited state diffusion, hybrid lead halide perovskite, photoluminescence, scanning near‐field microscopy

## Abstract

**Radiative recombination in thin films of the archetypical, high‐performing perovskites CH_3_NH_3_PbBr_3_ and CH_3_NH_3_PbI_3_** shows localized regions of increased emission with dimensions ≈500 nm. Maps of the spectral emission line shape show narrower emission lines in high emission regions, which can be attributed to increased order. Excited states do not diffuse out of high emission regions before they decay, but are decoupled from nearby regions, either by slow diffusion rates or energetic barriers.

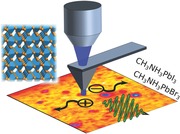

Organic–inorganic metal halide perovskite semiconductor‐based solar cells have recently reached power conversion efficiencies above 20%.^1^, ^2^, ^3^, ^4^, ^5^ In addition to their excellent photovoltaic properties, these materials also allow for high photoluminescence (PL) quantum efficiencies and optically pumped lasing.^6^, ^7^ Recently, bright perovskite‐based light‐emitting diodes^8^ have been demonstrated, which show the potential use of lead halide perovskites in a wider range of optoelectronic devices. Stranks et al. used PL quenching in vertical bilayer structures of lead halide perovskite with electron/hole acceptors to determine the diffusion length of photoexcited states in lead halide perovskites and obtained values of up to 1 μm. 9, 10 Recent estimations on single crystals of perovskites determine intrinsic diffusion lengths on the order of 100s of microns.^11^ Even diffusion lengths in the micron range would allow photoexcited carriers to sample large areas of the films before recombination, which raises the question of how the film microstructure changes recombination, and specifically whether spatial inhomogeneity influences the material and solar cell performance. The fabrication conditions of organic–inorganic lead halide perovskites affect the performance of optoelectronic devices^12^ and the optical properties of thin films.^13^, ^14^, ^15^, ^16^ The origin of these effects is not yet clear, but could be related to short‐range structural properties of lead halide perovskite thin films.

In the present work, we investigate the spatial recombination of photoexcited charges in thin methylammonium lead bromide and iodide films (CH_3_NH_3_PbX_3_, X = I, Br) by monitoring the spatially resolved PL. These two materials were chosen due to their use in photovoltaic cells^2^, ^17^, ^18^, ^19^ (CH_3_NH_3_PbI_3_) and light‐emitting diodes^8^ (CH_3_NH_3_PbBr_3_). We employ scanning near‐field optical microscopy (SNOM), which has been previously used to study local PL variations in nanostructured systems like polymer thin films,^20^ inorganic quantum structures,^21^ and polymer blends.^22^, ^23^ Recently, high spatial resolution techniques have been used to study charge extraction in hybrid lead halide perovskite cells and recombination in perovskite nanocrystals.^10^, ^24^, ^25^ Spectrally resolved PL emission maps of thin films on glass substrates were measured simultaneously with topography maps using a setup that combines SNOM optical excitation and atomic force microscopy (AFM). Local variations in emission intensity of several orders of magnitude are observed and localized hot‐spots are detected, which appear to act as radiative recombination centers in the films. The origin of these recombination centers is studied with time‐resolved PL and spectral analysis, which attributes the increased emission from these centers to the formation of long‐lived excited states in regions of high order and film quality.

Topography (a,c) and spectrally integrated PL intensity (b, d) maps are shown in **Figure** **1** for representative areas of CH_3_NH_3_PbBr_3_ and CH_3_NH_3_PbI_3_ thin films, respectively. All presented results were confirmed on several sets of films. Topography and PL maps were simultaneously measured at the same location using a SNOM setup (WITec alpha 300 s) with PL collection in transmission geometry. Excitation resolution was below 150 nm (Supplementary Figure 1, Supporting Information), and collection resolution was ≈400 nm (full details on spatial resolution in Experimental Section). The average thickness of the individual films was between 100 and 300 nm, in the range of the collection resolution, which minimizes effects of penetration depth or wave guiding. A continuous wave laser (*λ*_exc_ = 405 nm) was used for optical excitation of steady‐state PL. The PL intensity maps were normalized for differences in absorption by dividing the total detected PL per pixel with the fraction of absorbed light, which was calculated from the light transmission normalized to the maximum transmitted light intensity (Supplementary Figure 2a,b, Supporting Information)
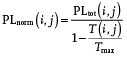
where *i* and *j* refer to one pixel in the map.

**Figure 1:**
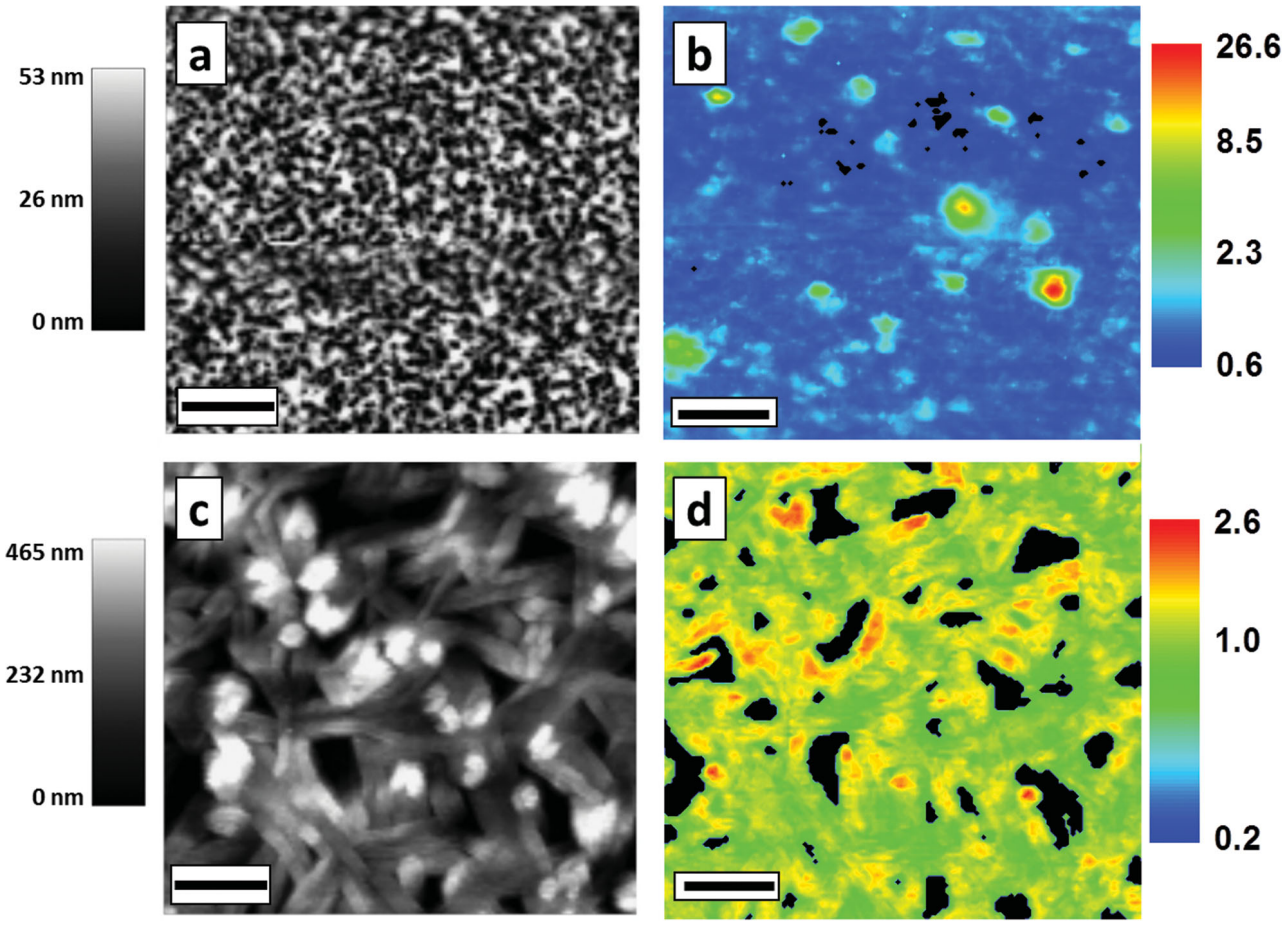
Topography (left), and map of the ratio between locally detected versus spatially averaged PL intensity (right) for spin‐coated a,b) methylammonium lead bromide CH_3_NH_3_PbBr_3_ and c,d) methylammonium lead iodide CH_3_NH_3_PbI_3_ perovskite thin‐films on glass. Films were excited using a continuous wave laser (*λ*_exc_ = 405 nm) in a scanning near‐field optical microscope (SNOM) setup. The PL emission was collected in transmission mode with confocal geometry. PL maps were corrected for small differences in absorption by measuring the transmitted excitation laser intensity. We observe localized spatial variations in PL intensity up to a factor of 26 (CH_3_NH_3_PbBr_3_) and 2.6 (CH_3_NH_3_PbI_3_), which do not show a correlation to features in topography. Maps have been collected over 130 by 130 data points. Length of scale bar = 4 μm.

In topography, the CH_3_NH_3_PbBr_3_ films appear homo­geneous (Figure 1a, Supplementary Figure 3a, Supporting Information) with a surface roughness of 17 nm and less than 5% of the film as voids. This demonstrates a significant enhancement in film quality compared to the standard PbBr_2_ precursor route, where over 70% of the film is as voids, which can be attributed to the use of Pb(CH_3_COO)_2_ precursor in our film fabrication.^26^, ^27^ Thin films of CH_3_NH_3_PbI_3_ were prepared following the standard, solution‐based fabrication route, using equimolar mixtures of CH_3_NH_3_I and PbI_2_. Their topography shows the typical micron sized needle‐like structures of as prepared spin‐coated films^12^, ^28^ with around 25% of voids and a surface roughness of 114 nm (Figure 1c, Supplementary Figure 3b, Supporting Information).

Spatially integrated emission spectra over the whole film show peaks at 535 nm (CH_3_NH_3_PbBr_3_) and 770 nm (CH_3_NH_3_PbI_3_), which is consistent with published spatially averaged film measurements.^29^, ^30^ Remarkably, local intensity variations are found in PL intensity maps for both perovskite types (Figure 1b,d). In CH_3_NH_3_PbBr_3_ films “hot spots” appear with up to 26 times higher PL intensity compared to the spatial average (Figure 1b) and variations in the PL intensity of several orders of magnitude are detected (Supplementary Figure 4a, Supporting Information). Local variations in the PL intensity are also seen in CH_3_NH_3_PbI_3_ films. However, in these films, areas of increased PL intensity are distributed more evenly over the film and only correspond to an increase in PL up to a factor of 2.6 compared to the spatial average (Figure 1d, Supplementary Figure 4b, Supporting Information). In particular, no enhanced emission is observed from the edge of crystallites. Additionally, light scattering and reflection could also lead to spatial modulation in the PL maps. However, in measurements taken in transmission and reflection on the same location, no differences in the PL patterns are observed, which indicates that scattering and reflection play a minor role (Supplementary Figure 5, Supporting Information). The localized emission features occur on length scales of several 100s of nanometers and do not correlate with structural features observed in topography (correlation coefficient between PL map and topography <0.2 for both perovskite types). The smallest observed features have diameters below 500 nm, which is smaller than published values of the charge carrier diffusion length in these materials.^9^ Since this value is close to our setup resolution of ≈400 nm even smaller features cannot be excluded.

Recombination kinetics can give further insights on the origin of the spatial intensity variations. The recombination dynamics of the photoexcited states are investigated with transient PL measurements in a confocal microscope setup with ≈250 nm spatial resolution (see Experimental Section), which allows us to selectively probe spatial regions of high and low PL intensity. The samples were excited with a 405 nm pulsed laser (PicoQuant) (pulse length ≈100 ps, excitation fluence 2 μJ cm^−2^ pulse^−1^, repetition rate 20 MHz for CH_3_NH_3_PbBr_3_, and 2.5 MHz for CH_3_NH_3_PbI_3_). Spectrally integrated PL was detected with an avalanche photodiode (IDQuantique ID100) and time‐correlated single‐photon statistics were recorded with a timing resolution of 150 ps (quTools quTau). Both perovskite types show slower recombination kinetics in regions of high emission intensity, compared to regions of lower intensity (**Figure** **2**a,b). Quantitative values for the change in initial recombination rates in CH_3_NH_3_PbBr_3_ and CH_3_NH_3_PbI_3_ samples were obtained from the characteristic lifetime, which is defined by the decay of the normalized PL intensity to 1/e. Multi‐exponential fits are shown as drawn lines as a guide to the eye. We use the characteristic lifetime to extract values for the initial decay rates at the employed low excitation densities, even though the underlying radiative recombination mechanism is of bimolecular nature, i.e., free charge carrier recombination.^31^, ^32^, ^33^ The insets show the decay of the PL at longer time scales. We point out that the longer fits in these figures represent tail fits, which only address a fraction ≤10% of the initial excitations. The intensities used here (≈5 × 10^16^ cm^−3^ peak density in transient measurements and ≈8 × 10^16^ cm^−3^ steady‐state excitation density in SNOM PL map measurements) are below those associated with photoinduced changes, such as bleaching and higher‐order recombination processes like Auger recombination.^34^ **Table** **1** summarizes the extracted lifetime values in two regions of high and low intensity. The errors in the extracted lifetime values represent a statistical uncertainty in the 1/e value of one standard deviation. The lifetimes show a ≈170% increase in high‐intensity regions for CH_3_NH_3_PbBr_3_ and about 150% increase for CH_3_NH_3_PbI_3_. Figure 2c–f show confocal PL intensity maps (c,d) and extracted lifetime values (e,f) for CH_3_NH_3_PbBr_3_ (left) and CH_3_NH_3_PbI_3_ (right) at different positions in a film. Spatial variations in emission lifetime quantitatively follow the absolute changes in emission intensity. This indicates that the changes in emission intensity are due to a faster recombination of carriers in low‐intensity regions. This suggests the presence of additional non‐radiative recombination channels in these areas, which compete with radiative recombination. Such an effect can be induced by differences in local defect density or material properties, which will be discussed below.

**Table 1:** Time constants of PL decays determined by the drop of emission intensity to 1/e for CH_3_NH_3_PbBr_3_ and CH_3_NH_3_PbI_3_ samples. Longer lifetimes are found in regions of high PL intensity compared to regions of low PL intensity for both hybrid lead halide perovskite types

	τ(1/e) [ns] High PL intensity region	τ(1/e) [ns] Low PL intensity region
CH_3_NH_3_PbBr_3_	1.34 ± 0.02	0.49 ± 0.01
CH_3_NH_3_PbI_3_	9.68 ± 0.07	3.85 ± 0.13

**Figure 2:**
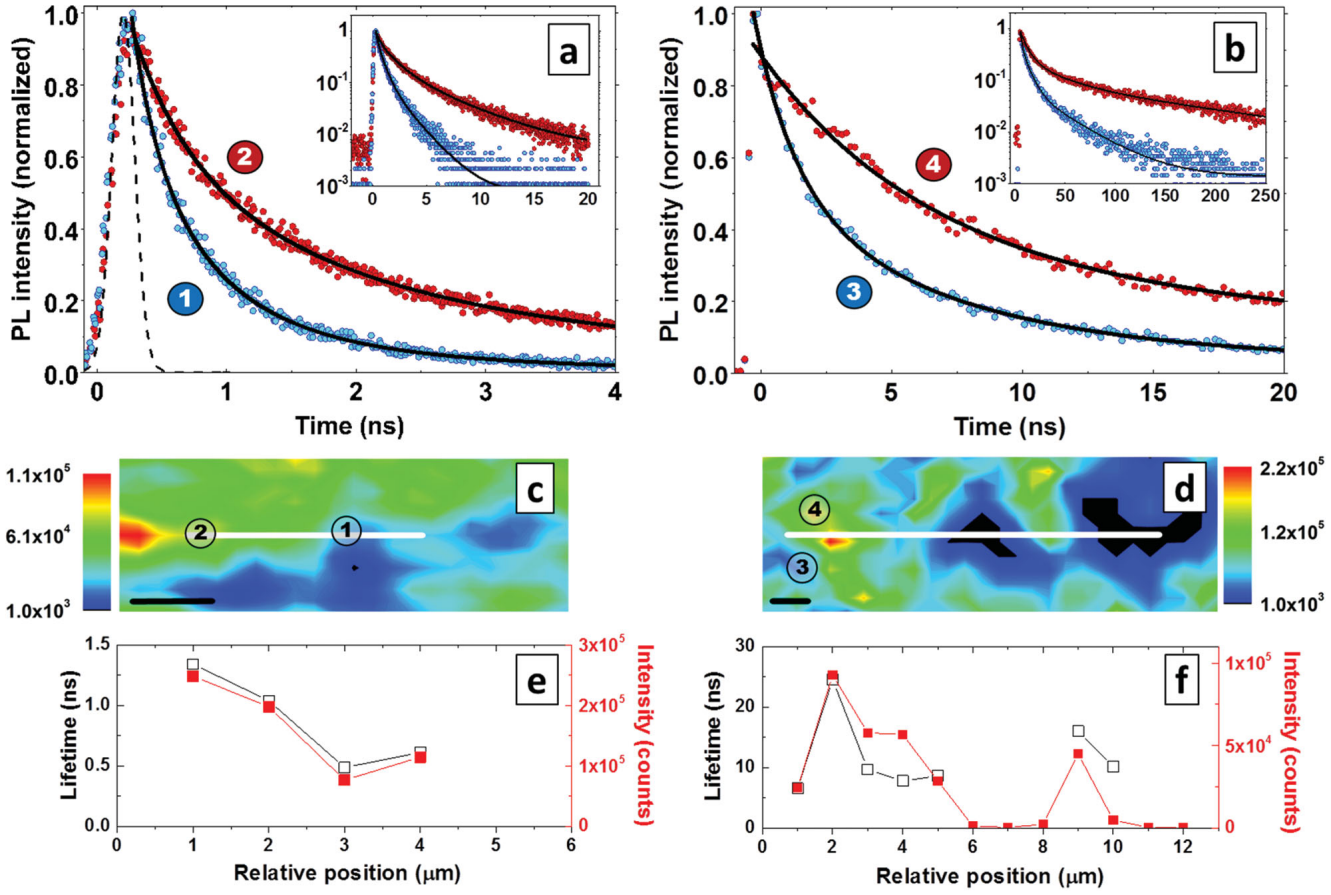
Representative transient PL decays taken in spatial regions of high (red) and low (blue) photoluminescence intensity, measured in a confocal microscope configuration with ≈250 nm resolution for a) CH_3_NH_3_PbBr_3_ and b) CH_3_NH_3_PbI_3_ samples on glass. The dotted line in Figure 2a shows the instrument response function. Insets in (a) and (b) shows the transient PL decays on logarithmic scale for longer time delays. The positions at which the decays are taken are indicated in the spatial emission maps of c) CH_3_NH_3_PbBr_3_ and d) CH_3_NH_3_PbI_3_. The black lines in (a,b) are fits to a bi‐exponential decay as a guide to the eye. The spatial variation in the extracted emission lifetimes and PL intensity values is shown in (e,f) for several positions along the white lines drawn in the PL maps (c,d). The error bars in the extracted emission lifetimes shown in (e,f) are smaller than the data points. Length of scale bar is 1 μm.

Hot‐spots in emission could be explained by highly localized recombination centers with smaller non‐radiative rates that would collect diffusing excitations from micron‐scale regions around them. In order to investigate this possibility, PL maps of CH_3_NH_3_PbI_3_ were measured in a confocal microscope geometry (100× objective, NA = 0.95) with a variable offset between excitation and detection focus. The films were prepared from Pb(CH_3_COO)_2_ precursor, which reduced the number of voids. PL was detected in transmission mode with a 40× objective (NA = 0.6) and detected using a spectrometer fitted with a CCD detector. Three PL maps are taken on the same area of the film: one with excitation and collection objective in alignment, which was found by maximizing the PL, and two with the collection being offset by 2 and 5 μm (Supplementary Figure 6, Supporting Information). PL maps with zero offset show comparable spatial variations in PL intensity as observed for CH_3_NH_3_PbI_3_ films from PbI_2_ precursor in Figure 1. As the collection objective is moved out of alignment with the excitation focus, the PL intensity decreases by a factor of five for an offset of 2 μm and a factor of ten for on offset of 5 μm. This relates to a relative decrease in emission of up to 85% (2 μm) and 94% (5 μm) compared to the PL in aligned position, without offset (Supplementary Figure 7, Supporting Information). In particular, no regions are detected, which maintain high PL emission, which would be a sign of long‐range excitation migration into “hot spots.” This does not match the scenario in which the high‐emission domains act as a very localized recombination center, as in that case it would be expected to observe the high PL regions even with an offset in detection alignment.

Representative PL spectra averaged over regions of high (red) and low (blue) PL intensity are shown in **Figure** **3** for CH_3_NH_3_PbBr_3_ (a) and CH_3_NH_3_PbI_3_ (b). No significant changes are detected in the low energy tail of the spectra, however the spectra at low PL intensity locations are broadened towards higher energies (lower wavelengths). We quantify this effect by monitoring the change in full width half maximum (FWHM) of the spectra. In CH_3_NH_3_PbBr_3_ the FWHM increases above 20 nm in regions of low PL intensity, while in CH_3_NH_3_PbI_3_ values above 35 nm are found. Values for the FWHM are extracted from the PL data in Figure 1b,d and are used to map the spatial distribution of the FWHM values, with PL intensity maps overlayed as red contour lines (Figure 3c,d). Lower FWHM values are mostly found in regions of increased PL intensity. The differences in FWHM between high and low emisison regions relate to energy differences of ≈35 meV for CH_3_NH_3_PbBr_3_ and ≈20meV for CH_3_NH_3_PbI_3_, which follows the observed trend in PL intensity. In spatial elemental distribution maps obtained from STEM‐EDX (scanning transmission electron microscopy–energy‐dispersed X‐ray spectroscopy) variations in the elemental weight ratio below ±5% compared to the average are observed (Supplementary Figure 8, Supporting Information), which would not lead to the observed shifts in emission.^19^, ^29^, ^30^ We further note that the spectral broadening cannot be explained by an increased excitation density in the high emission regions, since this would lead to the opposite effect, i.e., band filling and a tailing of the spectrum towards high energies in high emission regions. The change in FWHM may be explained by a higher crystallinity in the high intensity regions, which leads to changes in the energy level distribution. We therefore interpret the observed changes in the PL spectra as changes due to spatial differences in crystallinity. This interpretation is in agreement with high‐resolution TEM images, which show adjacent domains with different crystal orientation on a length scale of 10s of nanometers in these polycrystalline films (Supplementary Figure 9, Supporting Information). Further investigation of the localized high PL emission regions and the interfaces between these regions with more specialized techniques, i.e., spatially resolved electron diffraction or stimulated emission depletion microscopy should provide further details on the properties of these high‐emission domains.

**Figure 3:**
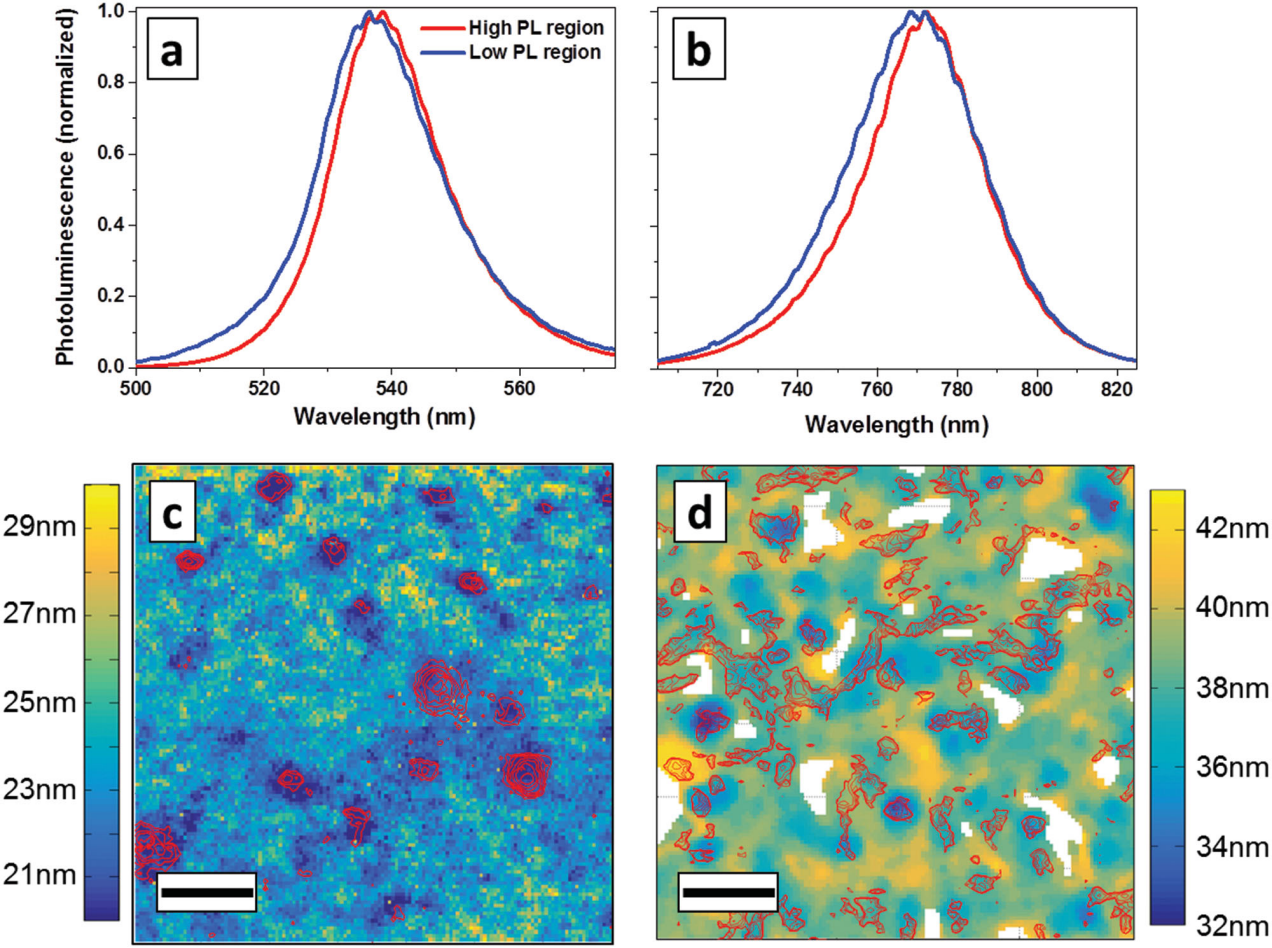
Representative PL emission spectra averaged over regions of high and low photoluminescence intensity, extracted from the absolute PL intensity data used for Figure 1, for a) CH_3_NH_3_PbBr_3_ and b) CH_3_NH_3_PbI_3_ samples. The spatial distribution of the photoluminescence emission peak full width half maximum (FWHM) was determined from locally recorded PL spectra for c) CH_3_NH_3_PbBr_3_ and d) CH_3_NH_3_PbI_3_ samples, respectively, with contour lines of high intensity PL regions as overlay (red). White areas represent voids in the film. We find spatial variations in FWHM and find lower FWHM values in regions of increased PL intensity. Length of scale bar = 4 μm.

Our results demonstrate that thin films of methylammonium lead halide perovskite exhibit spatially localized regions of high PL emission with sizes ≈500 nm, which show up to three times increased excited states lifetimes. While spatial PL intensity variations might be generally expected in a polycrystalline film with macroscopic disorder on a micrometer scale, it is surprising that these can be as localized as in our observations, in particular in light of the published long carrier diffusion lengths. In particular, our results indicate that regions with lower PL intensity and faster non‐radiative decay do not affect nearby high intensity regions; excited states generated within a “hot‐spot” region do not diffuse within their lifetime to the low‐intensity surrounding regions with higher non‐radiative decays, and are quenched there, since this would result in more uniform, low PL emission without localized “hot‐spots.” This suggests that high‐intensity regions are electronically decoupled from regions with fast non‐radiative decay channels, either by slow diffusion or energetic barriers, which prevent excited states to move effectively in a lateral direction from one region to the other. Radiative recombination appears to be affected by lateral carrier diffusion on a different length scale than the long vertical diffusion length scale that has been inferred from PL quenching or cross‐sectional electron‐beam induced current (EBIC) measurements.^9^, ^10^ We observe strongly reduced PL lifetimes in bilayer samples with PCBM charge acceptors (Supplementary Figure 10, Supporting Information), which supports the comparability of our results with previous work. Our results can be reconciled with previous measurements, if one considers the strong contribution of the high PL regions to the spatially averaged emission and a potential anisotropy of the in‐plane and out‐of plane diffusion in the film. High PL regions with high structural order could extend through the whole thickness of the film enabling long vertical diffusion lengths, while in‐plane diffusion of photoexcited states out of these regions may be restricted by their lateral size and energetic barriers associated with structural/grain boundaries. These radiative recombination centers show consistently narrower PL peaks, which can be attributed to lower energetic disorder. We have very recently become aware of a similar observation of spatially inhomogeneous photoluminescent properties in films of CH_3_NH_3_PbI_3_(Cl), which are interpreted in terms of spatial fluctuations of the iodine:chlorine ratio.^35^ Our results reported here on both I‐ and Br‐based perovskites that do not contain any Cl suggest that the observation might be more general and may not originate from fluctuations of elemental composition. It has been found that the presence of chloride aids nucleation,^15^ which would allow to reconcile the previous report^35^ with our findings and supports our more general interpretation that high‐intensity regions on perovskites are connected with higher crystallinity. The slightly red‐shifted and broader emission in the low PL regions in both I‐ and Br‐based films, which is consistent with the effect observed in ref.^35^ might be a general trend in those films. We interpret the high luminescent regions as areas of enhanced structural order and potentially increased crystallinity. The observed localized emission proposes that lead halide perovskites can show intrinsically benign material properties, with defective regions not affecting regions of superior semiconducting quality. The prospect of expanding the observed high quality regions to larger areas of the film provides a strategy to improve further the PLQE of these materials beyond the values reported to date^6^, ^8^ and to fabricate materials and devices with increased optoelectronic performance.

## Experimental Section

*Film Preparation*: Methylammonium iodide (CH_3_NH_3_I) and methylammonium bromide (CH_3_NH­_3_Br) synthesis are described elsewhere.^1^, ^30^ Equimolar mixtures of CH_3_NH_3_I and lead iodide (PbI_2_, Sigma–Aldrich, 99.999% pure) were dissolved in equimolar ratios in *N,N*‐dimethylformamide (DMF) in 40 wt% concentration. CH_3_NH_3_PbI_3_ thin film was spun in a nitrogen‐filled glove box onto quartz substrates at 2000 RPM for 45 s followed by 45 min thermal annealing at 100 °C inside the glove box. For the lead acetate (Pb(CH_3_COO)_2_)‐based perovskite thin‐film fabrication: 3:1 molar stoichiometric ratios of CH_3_NH_3_(Br, I) and Pb(CH_3_COO)_2_ (Sigma–Aldrich, 99.999% pure) were made in DMF in 20 wt% concentration. These solutions were spun inside a nitrogen filled glove box on quartz substrates at 2000 RPM for 60 s followed by 5 min of thermal annealing at 100 °C in air to form thin films. The prepared films show very low scattering as visible in Supplementary Figure 11 (Supporting Information).

*SNOM PL*: The combined PL and topography detection system are based on a WITec alpha 300 s SNOM. The excitation source is a 405 nm cw laser (Coherent CUBE), which is fiber‐coupled into the microscope. After the laser passes through a 20× Nikon objective, it is focused onto the backside of the hollow SNOM tip. The evanescent near‐field from the SNOM tip is used as excitation (schematic view of the setup in Supplementary Figure 1, Supporting Information). Due to the excitation in the near field, the optical resolution is only determined by the size of the SNOM aperture (<150 nm in our experiments, confirmed with the Fischer pattern tests done by WITec). Distance between the tip and the sample is controlled using a highly focused deflection laser (980 nm), which is reflected from the SNOM tip and does not interfere with the excitation laser. The PL signal is collected in transmission mode with a 40× objective (NA = 0.6), giving the collection resolution of ≈390 nm (Supplementary Figure 12, Supporting Information), and detected using a spectrometer fitted with a CCD detector. The transmitted laser light is collected with the bottom objective and, by moving the bottom objective in X, Y, and Z direction, it is possible to maximize the detected transmitted intensity. A low‐pass filter with a cut‐off wavelength at 435 nm was fitted before the CCD detector to block the 405 nm excitation laser. Maps are recorded from areas which show typical film features and for each measurement spot, the full PL emission spectra are acquired. In order to acquire 2D spectral and topography maps, the X–Y piezo stage of the microscope is used for sample movement, which is controlled by the WITec ScanCtr Spectroscopy Plus software in which all the data during scans are collected too. All maps are recorded in atmosphere with controlled temperature (20 °C) and humidity (35%). Films are measured on the same day when fabricated, being directly transferred from the nitrogen‐filled glove box and measured. No degradation of the sample or decrease of the PL over the course of measurement (≈2 h) has been observed. In long‐time control‐measurements degradation occurred after several days. In order to correct PL for changes in light absorption, transmission light intensity maps are taken with a photon detector. Simultaneously topography maps are taken using the WITec alpha 300 s SNOM system in transmission, with a SNOM aperture size below 150 nm.

The presented results were reproduced for a total of six spots on five samples for iodide and six spots on four samples for bromide. On individual spots multiple measurements were taken with reproducible results. The samples for this study were prepared by four different people at several occasions over 6 months. Scans were repeated at the same spots to check for degradation and the same results were found.

*Transient PL Measurements*: For transient PL measurements, a PicoQuant pulsed laser (407 nm wavelength, pulse length ≈100 ps, repetition rate 20 MHz) was used in confocal microscope geometry (100× objective, NA = 0.95—giving≈250 nm spatial resolution). Spectrally integrated PL was detected in reflection mode with an avalanche photodiode (IDQuantique ID100) and time‐correlated single‐photon statistics were recorded with a timing resolution of 150 ps (quTools quTau).

*STEM/EDX*: The perovskite films were analyzed in a FEI Osiris Transmission Electron Microscope operated in scanning (STEM) mode at an acceleration voltage of 200 kV. This is equipped with Bruker's Super‐X EDX detectors and a high‐brightness electron gun (X‐FEG), resulting in high count rates for elemental mapping. Elemental maps were acquired over areas of 8 × 8 μm^2^ and analyzed using Hyperspy [http://hyperspy.org/], employing the principal components analysis routine to de‐noise the data. Beam‐induced effects on the sample were limited thanks to the high EDX collection efficiency (100 ms dwell time per pixel) and the data de‐noising process using principal components analysis; this has been verified by iterated acquisition on the same area; the feasibility of analytical STEM on this class of materials has been shown by other groups in previous publications.^36^, ^37^ Samples for TEM were prepared by transferring scratched perovskite films on a TEM grid.

## Supporting information

As a service to our authors and readers, this journal provides supporting information supplied by the authors. Such materials are peer reviewed and may be re‐organized for online delivery, but are not copy‐edited or typeset. Technical support issues arising from supporting information (other than missing files) should be addressed to the authors.

Supplementary
