# Supplementary material for: Local Versus Long‐Range Diffusion Effects of Photoexcited States on Radiative Recombination in Organic–Inorganic Lead Halide Perovskites
Source: Adv Sci (Weinh). 2015 Jul 14;2(9):1500136. doi: 10.1002/advs.201500136 (PMC5115383; doi:10.1002/advs.201500136)
Supplement: Supplementary file 1 — Supplementary [file ADVS-2-0d-s001.pdf]

Copyright WILEY-VCH Verlag GmbH & Co. KGaA, 69469 Weinheim, Germany, 2015.

ADVANCED SCIENCE
Open Access

Supporting Information

for *Adv. Sci.*, DOI: 10.1002/advs. 201500136

Local Versus Long-Range Diffusion Effects of Photoexcited States on Radiative Recombination in Organic–Inorganic Lead Halide Perovskites

Milan Vruc´inic´, Clemens Matthiesen, Aditya Sadhanala, Giorgio Divitini, Stefania Cacovich, Sian E. Dutton, Caterina Ducati, Mete Atatüre, Henry Snaith, Richard H. Friend, Henning Sirringhaus, and Felix Deschler**

Copyright WILEY-VCH Verlag GmbH & Co. KGaA, 69469 Weinheim, Germany, 2013.

Supporting Information: Local versus long-range diffusion effects of photoexcited states on radiative recombination in organic-inorganic lead halide perovskites

Milan Vrućinić[1], Clemens Matthiesen[1], Aditya Sadhanala[1], Giorgio Divitini[2], Stefania Cacovich[2], Sian E. Dutton[1], Caterina Ducati[2], Mete Atatüre[1], Henry Snaith[3], Richard H. Friend[1], Henning Sirringhaus[1*], and Felix Deschler[1*]

[1]Cavendish Laboratory, University of Cambridge, JJ Thomson Avenue, Cambridge CB3 0HE, United Kingdom

[2]Department of Materials Science and Metallurgy, University of Cambridge, 27 Charles Babbage Road, Cambridge CB3 0FS, United Kingdom

[3]Department of Physics, Clarendon Laboratory, University of Oxford, Parks Road, Oxford, OX1 3PU, United Kingdom

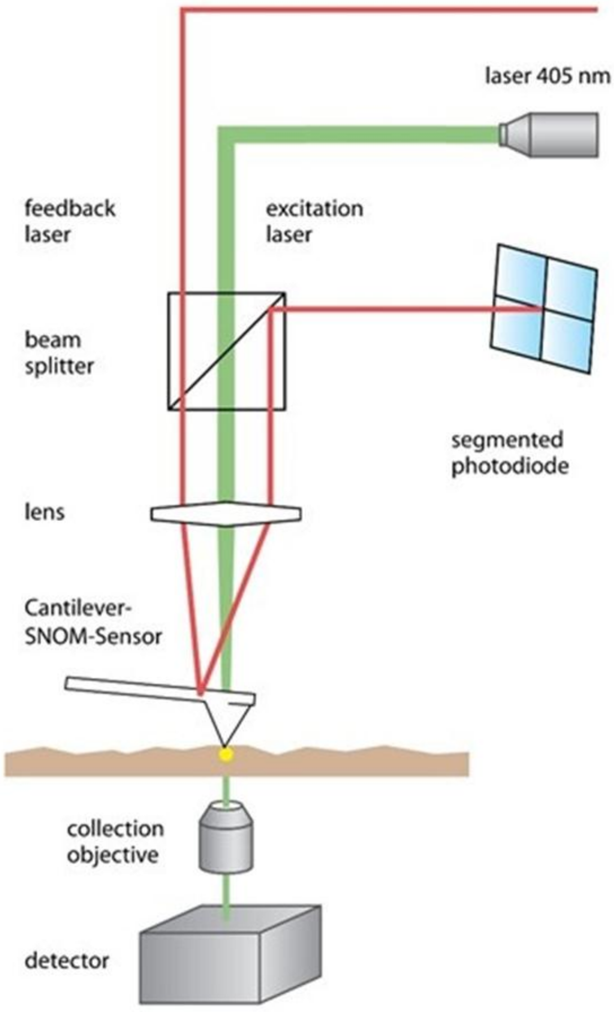

Supplementary Figure 1: Schematic view of the SNOM PL setup working in transmission. The excitation source is a 405 nm continuous wave laser (Coherent CUBE) which is fiber-coupled into the microscope. After the laser passes through a 20x Nikon objective it is focused onto the backside of the hollow SNOM tip, collected from the bottom 40x objective and detected using a spectrometer fitted with a CCD detector. Excitation light is filtered from the detected PL with a longpass filter.

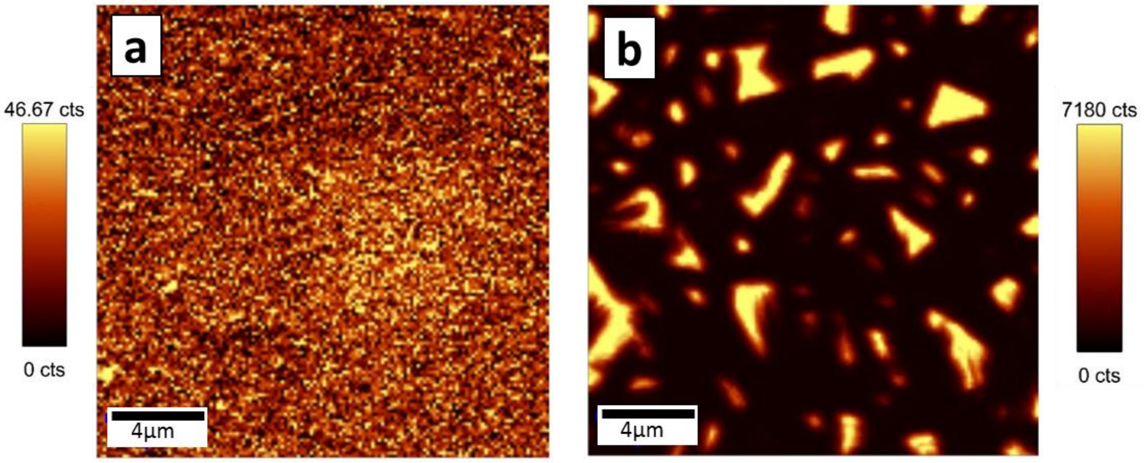

Supplementary Figure 2: Transmitted light intensity maps for (a) $CH_3NH_3PbBr_3$, and (b) $CH_3NH_3PbI_3$ by detecting the transmitted near field laser excitation through the film with confocal collection setup. The mapping was done with a continuous wave laser as probe light (405nm, Coherent LaserCube) in near-field to match the excitation conditions.

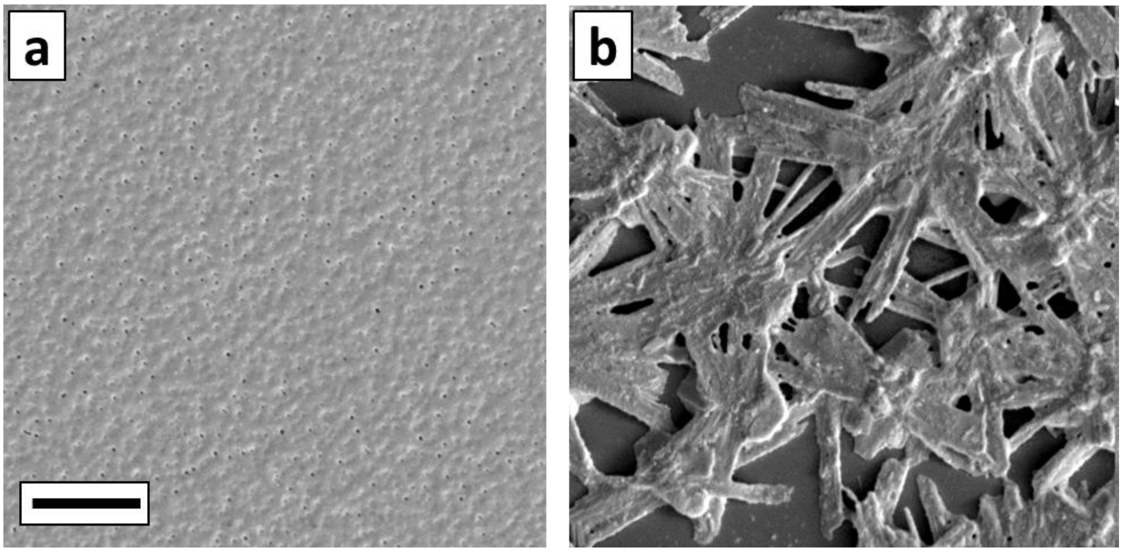

Supplementary Figure 3: SEM images for $CH_3NH_3PbBr_3$ (a) and $CH_3NH_3PbI_3$ (b) films on glass. Length of scale bar = 5µm.

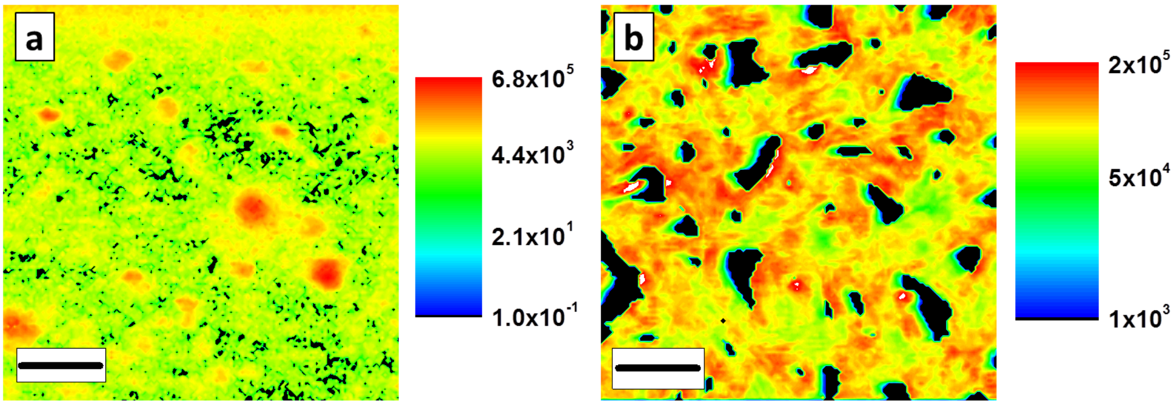

Supplementary Figure 4: Spectrally integrated SNOM PL intensity maps of spincoated pure methylammonium lead bromide $CH_3NH_3PbBr_3$ (a) and methylammonium lead iodide $CH_3NH_3PbI_3$ perovskite thin-films (b) on glass. The PL intensity maps were normalised for differences in absorption by dividing the total detected PL per pixel with the fraction of absorbed light, calculated from the light transmission normalised to the maximum transmitted light intensity (Eq. 1). Length of scale bar = 4μm.

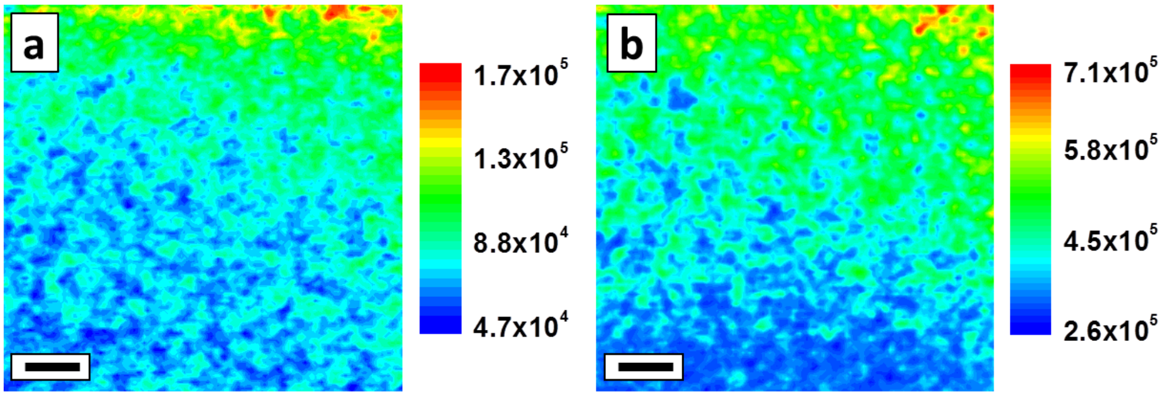

Supplementary Figure 5: Spectrally integrated PL intensity maps of spincoated methylammonium lead iodide $CH_3NH_3PbI_3$ perovskite thin-films (from lead acetate precursor) on glass taken in transmission (a), and reflection (b) mode. Length of scale bar = 4µm.

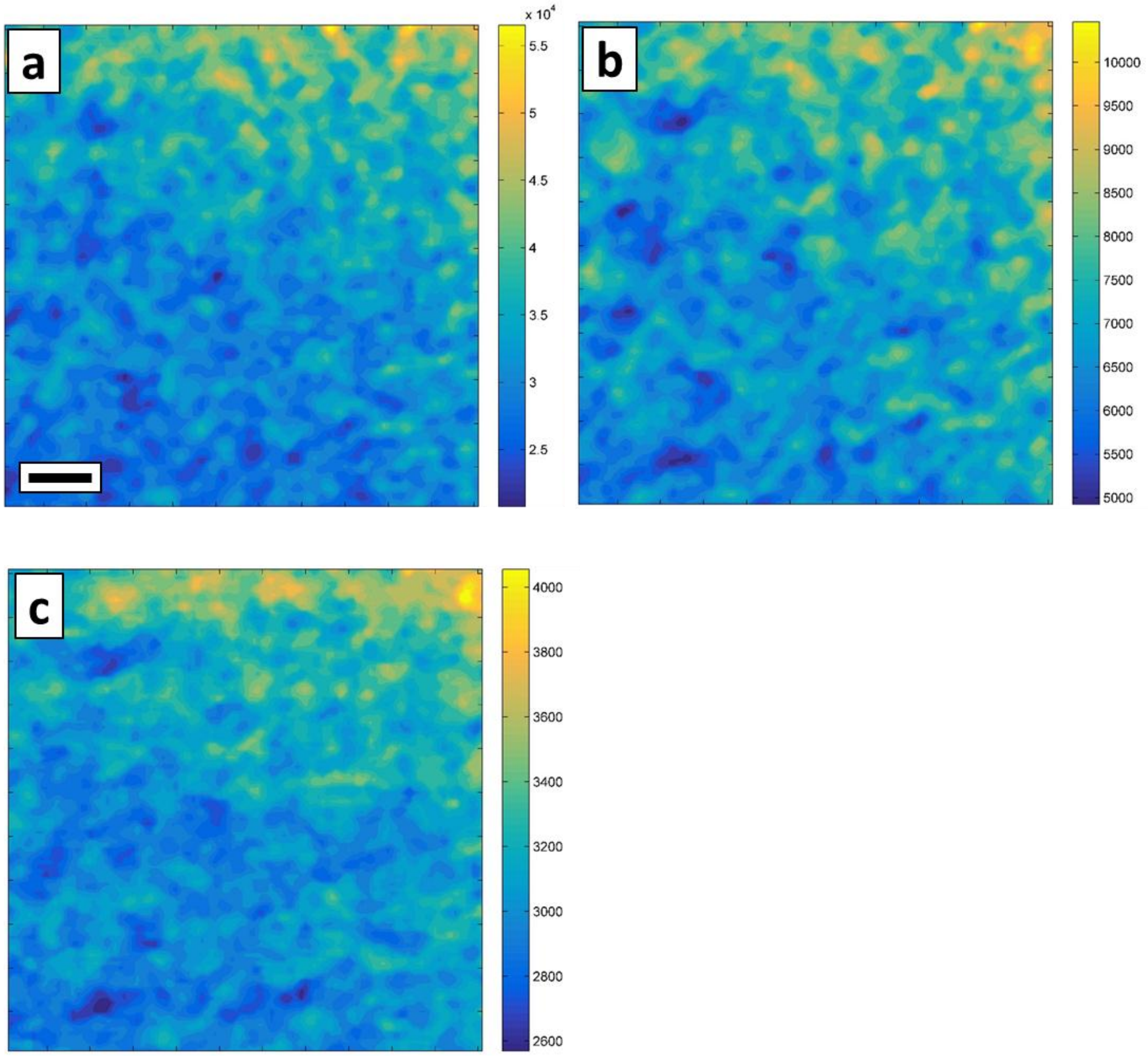

Supplementary Figure 6: Spectrally integrated PL intensity maps of spincoated pure methylammonium lead iodide $CH_3NH_3PbI_3$ perovskite thin-films on glass with aligned excitation and detection focus (zero offset) (a), and lateral offset of 2 µm (b) and 5 µm (c) between excitation and detection. Length of scale bar = 4µm.

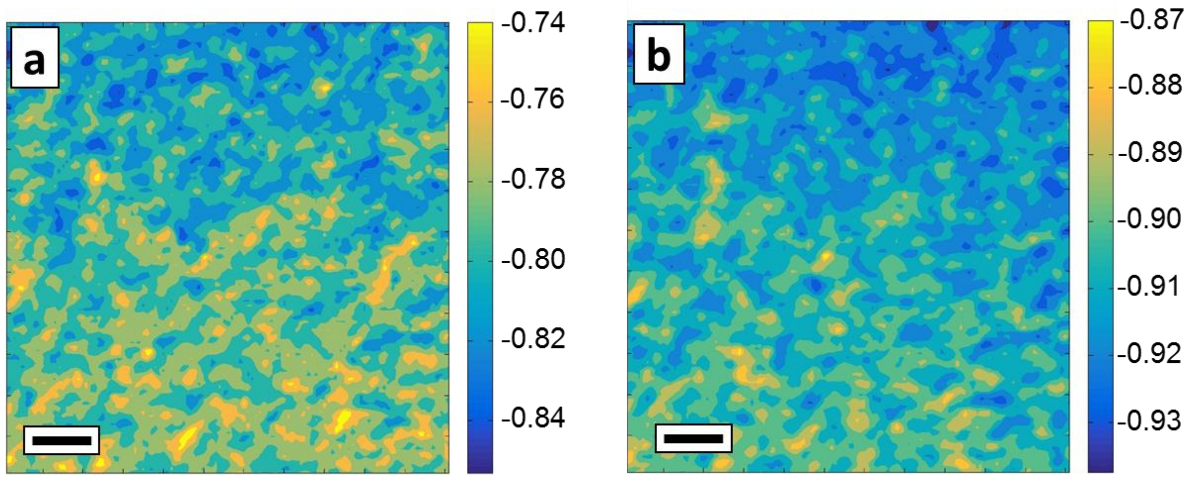

Supplementary Figure 7: Relative difference between spectrally integrated PL intensity maps for zero offset compared with PL maps at a lateral offset of 2 µm (a) and 5 µm (b). Corresponding absolute intensity maps are shown in Figure S5. Length of scale bar = 4µm.

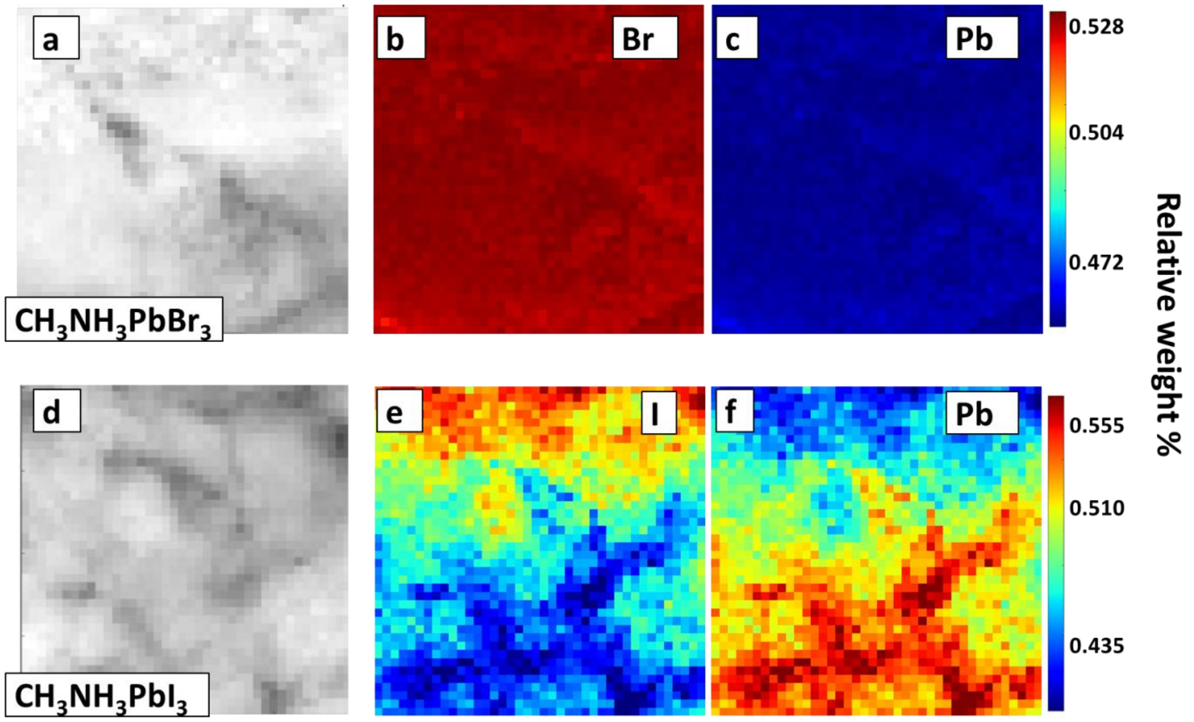

Supplementary Figure 8: STEM images of $CH_3NH_3PbBr_3$ (a) and $CH_3NH_3PbI_3$ (d) with the dimension of all images = 4µmx4µm. Samples were prepared by scratching films from glass substrates and transferring them onto TEM grids, which destroys the film large area structure, but retains the local elemental composition. Elemental maps were obtained by EDX on $CH_3NH_3PbBr_3$ samples for the elements Br (b) and Pb (c). Elemental maps on $CH_3NH_3PbI_3$ samples are shown for the elements I (e) and Pb (f). A homogeneous composition of the main structural elements is found in all samples with a variation in elemental composition below +/-5% with respect to average relative weight ratio. Similar results were obtained from samples directly spin-coated on TEM grids.

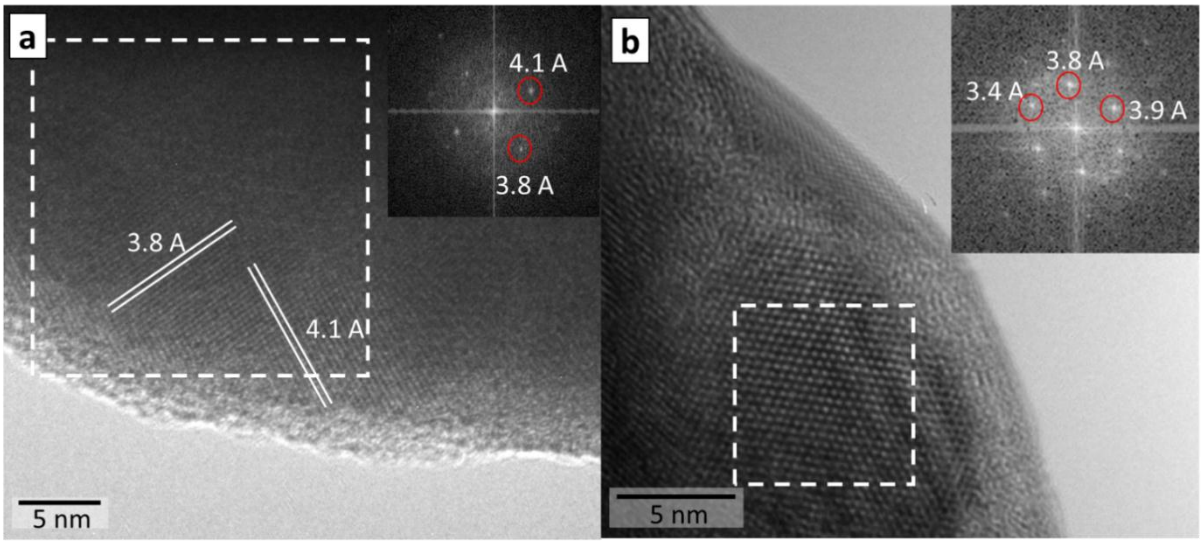

Supplementary Figure 9: HRTEM images of the $CH_3NH_3PbBr_3$ (a) and $CH_3NH_3PbI_3$ (b) samples. The FFTs of the areas in the dashed squares are reported in the insets, with the visible lattice spacings highlighted. The $CH_3NH_3PbBr_3$ specimen presents large crystalline domains; the $CH_3NH_3PbI_3$ sample is polycrystalline.

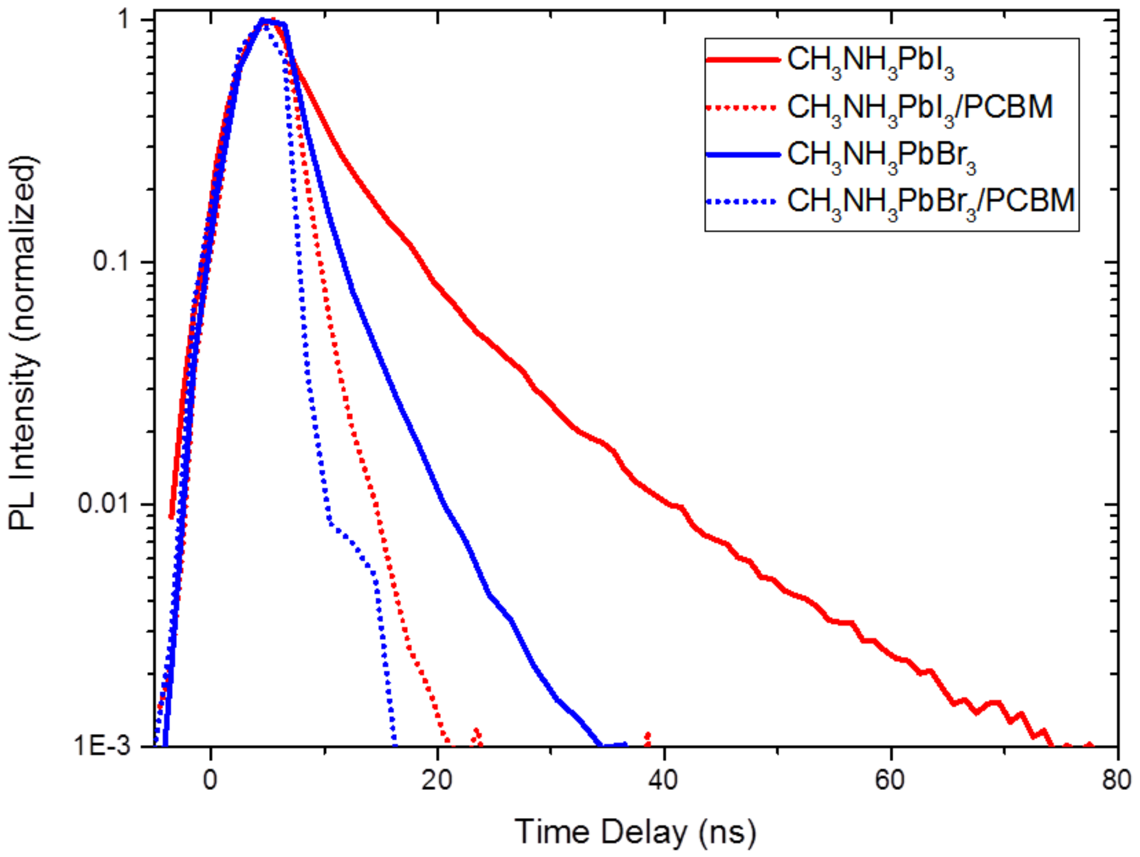

Supplementary Figure 10: Normalized photoluminescene kinetics of pristine films of $CH_3NH_3PbI_3$ and $CH_3NH_3PbBr_3$ and bi-layers with PCBM charge acceptor layer. Excitation with laser pulses (100 fs) at 400 nm with 1kHz repetition rate and fluence of ~$1x10^{13}/cm^3$. The PL decays of films with charge acceptor show reduced lifetimes close to the temporal resolution of the setup (~5ns).

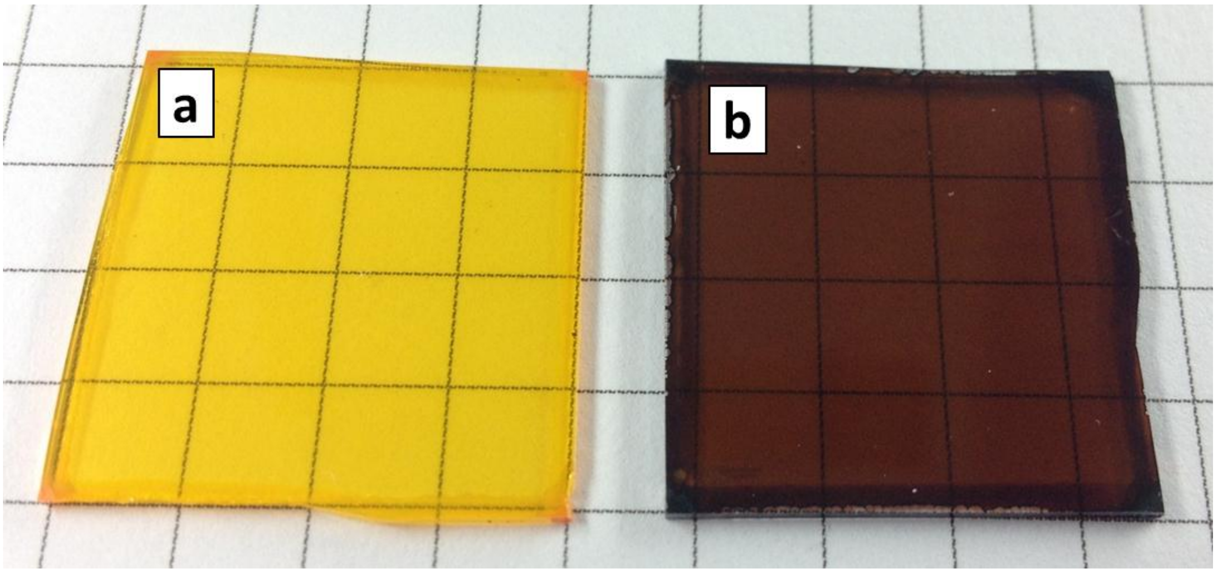

Supplementary Figure 11: Image of spincoated pristine methylammonium lead bromide $CH_3NH_3PbBr_3$ (a) and methylammonium lead iodide $CH_3NH_3PbI_3$ perovskite thin-films (b) on glass. The size of the samples is ~2x2cm. The pattern is clearly visible through the film, which indicates low visible light scattering in the samples.

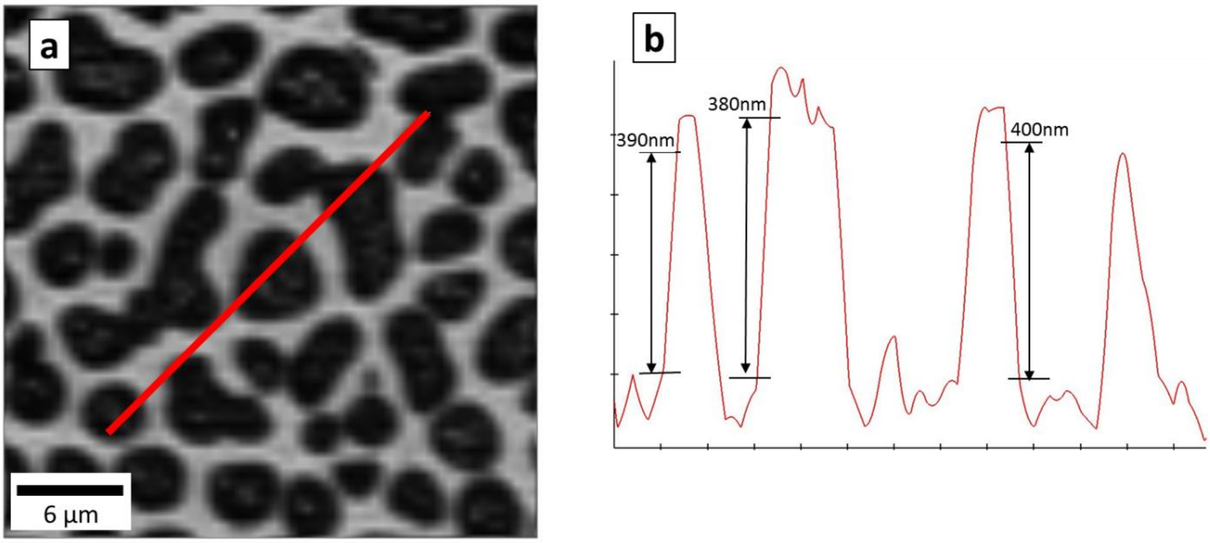

Supplementary Figure 12: Photoluminescence spatial maps of PFB:F8BT blend test samples [1, 2] taken in SNOM configuration of the setup as described in the Experimental Section (a). From the map a cross section (red line) is selected and used for further analysis (b). As resolution we use the 10-90 criterion on an edge which gives information about the steepness of the edge. We measure the distance from the point with 90 % intensity to the point with 10 % intensity (or vice versa). This distance gives a resolution of ~390nm.

References

[1] D. C. Coffey, D. S. Ginger, Nat Mater 2006, 5, 735.
[2] T. J. K. Brenner, C. R. McNeill, The Journal of Physical Chemistry C 2011, 115, 19364.
